# What can we learn from studying control arms of randomised VAW prevention intervention evaluations: reflections on expected measurement error, meaningful change and the utility of RCTs

**DOI:** 10.1080/16549716.2020.1748401

**Published:** 2020-04-27

**Authors:** Rachel Jewkes, Andrew Gibbs, Esnat Chirwa, Kristin Dunkle

**Affiliations:** Gender & Health Research Unit, South African Medical Research Council, Cape Town, South Africa

**Keywords:** Randomised controlled trials, non-differential misclassification, proportionate change, sample size, gender-based violence, violence against women, bias, regression towards the mean

## Abstract

**Background**: Randomised controlled trials (RCTs) are a gold standard for evaluations in public health, economics and social sciences, including prevention of violence against women (VAW). They substantially reduce bias, but do not eliminate measurement error. Control arms often show change, but this is rarely systematically examined.

**Objective**: We present a secondary analysis of data from the control arms of evaluations of VAW prevention programming to understand measurement variance over time, factors that may systematically impact this and make recommendations for stronger trial design and interpretation.

**Methods**: We examine data from six RCTs and one quasi-experimental study, all of which used comparable measures. We look at change over time among control participants in prevalence of physical intimate partner violence (IPV), sexual IPV, and severe physical/sexual IPV, by participants’ gender and study design (cohort vs. repeat cross-sectional).

**Results**: On average, repeated assessments of past year IPV varied by 3.21 (95%Cis 1.59,4.83) percentage points for the studies with no active control arms. The prevalence at endline, as a proportion of that at baseline, on average differed by 17.7%. In 10/35 assessments from 4/7 studies, the difference was more than 30%. We did not find evidence of the Hawthorne effect or repeat interview bias as explanations. Our findings largely supported non-differential misclassification (measurement error) as the most likely error and it was a greater problem for men.

**Conclusion**s: Control arms are very valuable, but in VAW research their measures fluctuate. This must be considered in sample size calculations. We need more rigorous criteria for determining trial effect. Our findings suggest this may be an absolute change in prevalence of 7% and proportionate change of 0.4 or more (especially for studies in populations with lower IPV prevalence (<20%)). More elaborate pre-defined outcomes are necessary for determining impact (or possible harms) of VAW prevention interventions.

## Background

Violence against women (VAW) is a major violation of women’s human rights and undermines their health and general social and economic development. The Sustainable Development Goals’ (SDGs) target of ending VAW stems from recognition of these harms, and high-quality research on VAW prevention is crucial for informing and enabling progress towards this goal. Work in this regard is growing rapidly; a recent overview of the field identified 96 evaluations of VAW prevention interventions using RCTs or quasi-experimental studies with control arms, 65 from low- and middle-income countries [1]. However, this same review of the field also highlighted the need for reflection on methodological issues in this research, not least because many of the studies were impacted by important flaws in their design and data analysis. The review itself can be criticised for privileging particular forms of research over others, as it did not include studies that lacked control arms or consider qualitative evaluations. This is a common practice in reviews, as well as in primary research, but is an increasingly contested. RCTs are touted as a ‘gold standard’, but this position is not without challenge [2].

Operational criticisms of RCTs are that they are complex and very expensive to do well. The push from multiple sources for RCT-type evidence also means that RCTs may be used too readily in evaluating interventions that lack the foundational research needed to optimise them before evaluations with RCTs or that the interventions evaluated are fundamentally incompatible with the methods of RCTs, resulting in time and resources wasted on studies with predictably null findings. RCTs have also been criticised for often being unfair to controls (except in delayed intervention or stepped wedge trials, where controls receive the intervention at some point). Additionally, they are usually inflexible, constraining intervention delivery and preventing adaptive programming. The result is ungeneralizable results from testing interventions in circumstances that bear little resemblance to how programmes are implemented in ‘real world’ settings [2–4].

Proponents of RCTs argue that they are the most rigorous evaluation methodology because they reduce multiple forms of bias, i.e. selection bias, confounding and information bias [5]. Trial populations may differ from the broader population in ways that can impact response to an intervention, i.e. demonstrate selection bias. In an RCT, random allocation of participants (or sites/clusters) to the different arms, where possible in small blocks, evenly distributes participants, with their various characteristics between the study arms. Confounding factors are associated with both an exposure of interest and the outcome for which this exposure is possibly a risk factor. One of the most common confounding factors in research is age, which is often related to health or other outcomes. If the RCT sample size is large enough, confounding factors should be randomly allocated between the arms, and examining the distribution of measured confounders by at baseline is an important test for this.

The third type of bias, information bias, results from inaccurate reports of whether a research participant has an exposure or outcome. The bias may operate systematically across participant sub-groups such as one arm of an RCT (resulting in differential misclassification), or by chance and therefore impact both RCT arms (in non-differential misclassification). For example, recall bias results in differential misclassification and is seen when disclosure is linked to the intervention provided in a study arm, e.g. if understanding more about VAW through a prevention intervention led to more participants disclosing experiences of abuse than in the control arm. A bias towards giving ‘pleasing’ responses may also result in differential misclassification and over-reporting an intervention’s impact on VAW if a desire to ‘please’ project staff causes participants say they no longer experience VAW when actually they do. Reporting period bias may impact both study arms. The latter may result in an apparently larger change in prevalence of the outcome in both arms between, e.g., baseline and midline, than between midline and endline. Reporting period bias is due to participants including in their report events that occurred outside the reporting period either because they forget the timing, or do not understand the importance of the precise period, possibly because they feel important events ‘must’ be of interest, irrespective of when they occurred (e.g. thus a severe assault that occurred 2 years ago might be included in response to a question about the past year, because it was so notable). Reporting period error may be a particular problem the first time participants are asked a question, as in cross-sectional research, and less so with repeated cohort study interviews. Differential misclassification can result in bias in any direction, depending on the circumstances and may be hard to identify.

Non-differential misclassification, which applies equally to both study arms, may stem from the wording of questions, circumstances of the data collection, the fact of observation, or be due to chance. Several authors writing about VAW research have drawn attention to the potential for bias in data collection and the need to reduce it or to ensure that it is non-differential in evaluations [6,7]. For example, interviewer effects are seen when an interviewer’s warm approach leads to more disclosure of sensitive exposures. These can be reduced by ensuring interviewers work equally in all trial arms, or by using ACASI, which also reduces social desirability bias. Interview context bias is important in sensitive research and must be addressed by having interviews conducted in a relaxed private environment. Non-differential misclassification results from poor question design when a question is quite hard to answer correctly (e.g. ’were you breastfed for six months or more?’) or is vague and likely to be randomly interpreted (‘have you ever been severely assaulted?’). The Hawthorne effect describes behavioural changes that occur just because a participant is being studied [8], essentially the questionnaire and questions lead to changes in behaviour. A further phenomenon that results in a non-differential error in reported prevalence is not actually a bias, but occurs when a first measure shows an extreme value and later measures show values much closer to the centre of the distribution [9]. This stems from the fact that all measures lie within a distribution, the dimensions of which are a product of both ‘actual’ levels of the measure and a degree of random variation. The latter is reduced by having a larger sample size [9].

Orthodoxy has it that non-differential misclassification biases study outcomes towards the null in studies with binary outcomes, although it may be away from the null in studies with more complex outcomes [10]. However, emerging research on VAW shows that for an outcome where misclassification overwhelmingly consists of under-reporting (rather than over-reporting), misclassification impacts prevalence of reports without necessarily resulting in bias towards the null [11].

The evolution of methodologies for evaluating VAW prevention interventions has to date focused more on some non-differential misclassification errors, such as interviewer effects and context, than others. There have been efforts to improve questionnaires used for measurement, but most of the research has involved survey populations, and an enduring difficulty stems from their being no true reference measure for outcomes in VAW research. Little is known about the reliability of the most commonly used measures of VAW in different populations or for men versus for women, notwithstanding some attempts to engage with this arena [12]. Furthermore, there is growing debate about whether the key outcome from VAW prevention trials should be total cessation of violence, lower frequency of violent acts, or non-initiation of violence, and whether all violence should be considered together or particular types privileged, or independently examined (such as physical and/or sexual IPV rather than emotional or economic IPV). Having multiple measures of VAW enhances the likelihood of examining the ‘right thing’ but also increases the risk of positive findings due to chance with multiple testing. Understanding the performance of VAW outcome measures and patterns of change supported by secondary outcomes is very important.

A control arm is presumed to experience all conditions of an RCT except the intervention and to, therefore, provide a comparison against which intervention effects can be isolated. It is therefore relied upon to reduce the impact of bias. However, the distribution of outcomes in the control arm is not often examined. We were not able to find a published paper that has critically reflected on this key element of evaluations across a series of related clinical trials. Despite its importance, and the fact that assumptions are made about control arms in sample size calculations, the behaviour of control arm participants is usually taken for granted, or if noticed to change, treated as study ‘noise’, rather than being worthy of discussion. Yet many evaluations of VAW-related interventions observe a considerable change in their control arms. Researchers often perceive the impact of control arm changes on their study power, as such changes are rarely accounted for in a sample size calculation, and may find themselves profoundly uncertain about whether to interpret control arm change a genuine impact of a low dose intervention, or as some form of Hawthorne effect or other impact of the research process.

The UK Aid-funded *What Works to Prevent Violence Against Women and Girls? Global Programme* provided a valuable opportunity to reflect on methodological issues in VAW evaluation research through analysis across the datasets of the multiple RCTs or quasi-experimental evaluations of VAW prevention interventions. In this paper, we present and discuss seven studies with control arms conducted with adult participants. We discuss four questions: What are the underlying trends in VAW among participants in the control arm? How much do the control participants change and does this depend on gender, study design, or type of IPV being measured? What is the proportionate change of VAW in the control arm? What can we learn for future VAW prevention intervention evaluation research and interpreting their findings?

## Methods

This paper presents a secondary analysis of data from 12 datasets arising from seven impact evaluations of VAW prevention programmes. They were conducted in six countries as part of the UKAID-funded, *What Works to Prevent Violence Against Women and Girls* (*What Works*) programme. Six of the evaluations were randomised controlled trials (RCTs) and the seventh was a quasi-experimental study with a control arm. Some of the studies had closed cohorts, where the same participants were followed across the trials and interviewed multiple times, and some of the studies involved interviewing repeated randomly selected cross-sections of the study site residents, in which case the individuals interviewed largely differed at baseline, midline and endline. We used data from the control arms of all studies. A summary of the methods of the evaluations, with a focus on the control arms, is presented in Table 1. All trials used frontline field staff of the same sex as the study participants and standardised field procedures between data collection rounds.

**Table 1.** Summary of the methods of the trials

Evaluation	Study design	Face to face/ ACASI	Participants	Control arm sample	Control arm intervention exposure	Papers
Stepping Stones Creating Futures, South Africa	2-arm RCT: treatment and delayed intervention control. Assessments: baseline, 12 and 24 months	ACASI	Cohort of men and women. Not recruited as couples	Baseline: 336 men, 338 women (17 clusters); Endline: 268 men, 285 women	None	[13,14]
Indashyikirwa couples, Rwanda	2-arm RCT: treatment and control. Assessments: baseline, 12 and 24 months	ACASI	Cohort of men and of women who were married/cohabiting couples	Baseline: 832 women, 830 men; endline: 818 women, 803 men	None	[15,16]
Indashyikirwa community, Rwanda	2-arm RCT: treatment and control. Assessments: baseline and 24 months	Both used (choice provided)	Residents in households in the districts of the study. Repeat cross-sectional data	Baseline and endline: 700 currently-partnered women, 700 men (14 clusters)	None	[15,16]
Change Starts at Home, Nepal	2-arm RCT: treatment and control. Assessments: baseline, 12 and 28 months	F to F	Household residents in the study districts. Repeat cross-sectional data	Baseline: 720 women; Endline: 717 women (18 clusters)	None	[17,18]
Women Empowerment Programme, Afghanistan	2-arm RCT: treatment and delayed intervention control. Assessments: baseline, 12 (data not presented) and 22 months’	F to F	Cohort of women	Baseline: 714 women (453 currently married); Endline: 537 (441 married)	None	[19,20]
Rural Response System, Ghana	2-arm quasi-experimental study: treatment and control. Assessments: baseline and 24 months	F to F	Households recruitment in 2 control districts. 10 male eas, 10 female. Repeat cross-sectional data	Baseline: 1075 men and 1047 women (20 eas); Endline: 1177 men and 1170 women	None	[21,22]
VATU, Zambia	2-arm RCT: treatment and delayed intervention control. Assessments: baseline and 12 months	ACASI	Cohort of couples (men and women). known problems of IPV and the man harmfully using alcohol	Baseline: 125 couples; endline: 114 women and 108 men	Weekly phone calls (or home visits) by study assessors asking questions on suicidal and homicidal ideation, current risk of IPV and child abuse. With safety plans for any identified risk	[23]

The studies all used very similar questions to assess their main IPV outcomes – experience of physical IPV, sexual IPV (data not collected in Afghanistan) and severe IPV in the past 12 months reported by partnered women, or self-reported perpetration by partnered men. Drawing questions from the World Health Organisation’s violence against women scale (as adapted for men) [24,25], a participant was classified as having experienced/perpetrated physical IPV if they responded positively (once, few times or many times) to any of the 5 items on physical IPV. A typical item was ‘In the last 12 months how many times did you hit your current or previous girlfriend or wife with a fist or with something else which could hurt her?’ Similarly, a participant was classified as having experienced/perpetrated sexual IPV if they responded positively to any of the three items on sexual IPV. A typical item was: ‘In the last 12 months, how many times have you physically forced your current or previous girlfriend or wife to have sex with you when she did not want to?’ The responses to these questions were never, once, few or many times. Severe IPV was assessed by combining the five physical and three sexual IPV questions (apart from Afghanistan where we only asked physical IPV questions). Participants were deemed to have experienced (women) or perpetrated (men) severe IPV if a participant responded positively to two or more items, or else responded: ‘few’ or ‘many’, to any single item from these eight questions.

All of the studies had appropriate approval from research ethics committees and all participants provided voluntary consent to participate in them. The details are provided in the study’s respective primary publications.

## Data analysis

For this analysis we consider the following questions in respect of the control arms:

- What was the change in percentage points between baseline and endline?
- If there was a larger change between baseline and midline, or midline and endline, what was this change in percentage points?
- What was the proportionate change between baseline and endline?
- If there was a larger change between baseline and midline, or midline and endline, what was this proportionate change?
- Did the patterns of change differ between men and women? Between cohort and repeat cross-sectional studies? Between different types of IPV?

A data set was generated with study, gender, type of IPV, and measure at each time point, from the original datasets. This was entered into Stata 15.0. For assessments made across all measures of IPV, a cluster variable was created for the study and clustering within a study was taken into account using Taylor linearization. We have not included the VATU, Zambia dataset in all of the assessments as it was the only study with an active control arm (rather than no intervention in the control arm during the main study) and it is reasonable to conclude that the assessments in the control arm may have been influenced by this.

## Results

### Change in prevalence of IPV in the control arms

Table 2 shows the prevalence of IPV experience or perpetration in the past 12 months, by type of IPV, in the control arm of seven different studies at the different time points at which women and men in the studies control arms were interviewed. There were considerable differences among the studies in the prevalence of physical IPV at baseline, ranging from 75.2% experiencing physical IPV reported by women in the VATU study in Zambia to 10.7% self-reported perpetration by men in Ghana. The prevalence of sexual IPV ranged from 69.6% (women in Zambia) to 8.5% (women in Ghana). The prevalence of severe IPV ranged from 81.4% (women in Zambia) to 16.8% (women from Afghanistan, but sexual IPV was not included) or 17.0% (men in Ghana, where severe IPV included both physical and sexual IPV).

**Table 2.** Prevalence of IPV in the control arm across studies, by IPV type, and quantum of change between time points

	Gender	IPV measure	Baseline	12-mths	24-mths	Change in percentage points baseline – endline	Largest change in percentage points between two time points	Proportionate change (baseline to endline)	Largest change as a proportion of baseline
Stepping Stones Creating Futures, South Africa	Women	Physical	60.1	49.3	54.4	−5.7	−10.8	−0.095	−0.180
		Sexual	30.2	32.1	35.4	5.2	5.2	0.172	0.172
		Severe	56.2	45.1	50.9	−5.3	−11.1	−0.094	−0.198
	Men	Physical	50.4	53.7	44	−6.4	−6.4	−0.127	−0.127
		Sexual	30.4	44	27.2	−3.2	13.6	−0.105	0.447
		Severe	46.6	49.5	40.7	−5.9	−5.9	−0.127	−0.127
Indashyikirwa couples, Rwanda	Women	Physical	33.1	34.4	32.2	−0.9	1.3	−0.027	−0.027
		Sexual	37.6	38.5	38.1	0.5	0.9	0.013	0.024
		Severe	41.1	43.5	42	0.9	2.4	0.022	0.058
	Men	Physical	22.8	19.4	16	−6.8	−6.8	−0.298	−0.298
		Sexual	19.6	17	15.6	−4	−4	−0.204	−0.204
		Severe	22.4	19.4	16.7	−5.7	−5.7	−0.254	−0.254
Change Starts At Home, Nepal	Women	Physical	20.4	12.9	16.0	−4.4	−7.5	−0.216	−0.368
		Sexual	23.9	15.8	23.0	−0.9	−8.1	−0.038	−0.339
		Severe	28.4	19.6	25.8	−2.6	−8.8	−0.092	−0.310
Women’s Empowerment Programme, Afghanistan	Women	Physical	23.9		26.6	2.7	2.7	0.113	0.113
		Severe	16.8		19.6	2.8	2.8	0.167	0.167
RRS, Ghana	Women	Physical	15.3		10.9	−4.4	−4.4	−0.288	−0.288
		Sexual	8.5		6.8	−1.7	−1.7	−0.200	−0.200
		Severe	17.6		11	−6.6	−6.6	−0.375	−0.375
	Men	Physical	10.7		12.6	1.9	−1.9	0.178	−0.178
		Sexual	16		19.8	3.8	3.8	0.238	0.238
		Severe	17		19.4	2.4	2.4	0.141	0.141
Indashyikirwa community Rwanda	Women	Physical	40.9		41.9	1	1	0.024	0.024
		Sexual	46		43.8	−2.2	−2.2	−0.048	−0.048
		Severe	50.9		49.7	−1.2	−1.2	−0.024	−0.024
	Men	Physical	22.4		24.1	1.7	1.7	0.076	0.076
		Sexual	16.7		17.3	0.6	0.6	0.036	0.036
		Severe	19.7		21.4	1.7	1.7	0.086	0.086
VATU couples study, Zambia	Women	Physical	75.2	47.4		−27.8	−27.8	−0.37	−0.37
		Sexual	69.6	46.5		−23.1	−23.1	−0.332	−0.332
		Severe	81.4	44.9		−36.5	−36.5	−0.552	−0.552
	Men	Physical	74.4	49.4		−25	−25	−0.336	−0.336
		Sexual	43.2	38.3		−5	−5	−0.115	−0.115
		Severe	73.7	39.0		−34.7	−34.7	−0.529	−0.529

Only seven datasets had an assessment 12 months after baseline presented in the table. Interviews at this time point were conducted in Afghanistan, but there were significant problems with the dataset due to inadequate identification of the cohort and so data are not presented. Overall the range of IPV prevalence at 12 months was narrower than at baseline, but there was no consistent pattern of difference between the two assessments by study. At the 12 months interview, the range of reports of physical IPV was 53.7% (men in South Africa) to 12.9% (women in Nepal). The range of sexual IPV was 46.5% (women in Zambia) to 15.8% (women in Nepal), and the range of severe IPV was from 49.5% (men in South Africa) to 19.4% (men in Rwanda couples).

Only 10 studies had an assessment 22–28 months post-baseline. At this time point the range of physical IPV was 54.4% (women in South Africa) to 12.6% (men in Ghana), and for sexual IPV was 38.1% (women in Rwanda couples) to 6.8% (women in Ghana) and for severe IPV was 50.9% (women in South Africa) to 11.0% (women in Ghana). Overall there was a change in the control arms and most commonly it was in the direction of lower reporting at endline (23/35 assessments), but there was no consistent pattern of change by study or type of IPV.

The percentage point change between baseline and endline varied between studies and types of IPV. The two datasets from the VATU study in Zambia were marked outliers in that, with the exception of men’s reports of sexual violence, all the reports were of substantial changes ranging from 23.1% to 36.5%. This was the one study that had an active control arm. For the studies that did not have an active control arm, across different measures of IPV, the change ranged between a reduction of 6.8 percentage points and an increase of 5.2 percentage points. Overall across all datasets in about 41% of measures (12/29), there was a higher prevalence of IPV reported at endline than baseline and in 59% (17/29) the endline was lower.

There were, however, studies without active controls where there was greater change either between baseline and midline, or midline and endline. Overall 12/28 (43%) changes were in the direction of this greater change, showing an increase in prevalence. The range for the greatest change was between an increase of 13.6 and 0.9 percentage points (men, sexual IPV in South Africa and in the Rwanda couples sample) and a negative change (reduction) of between 11.1 and 1.2 percentage points (women, severe IPV in South Africa and women, severe IPV in Rwanda community, respectively).

The proportionate change between baseline and endline ranged from an increase of 0.238 to 0.013 (both sexual IPV, men in Ghana and women in Rwanda couples), and a reduction of −0.375 to −0.024 (women, severe IPV Ghana and women in Rwanda community). The largest proportionate change was reported for sexual IPV by men in South Africa, which increased proportionately by 0.447 at the midline data point.

The VATU study had by far the largest absolute changes in five of the six measures; however, the range of proportionate change was a reduction of 0.552 to 0.115. The upper end of this range was the largest proportionate change, though there were several other measures from other studies that also showed a high proportionate change, i.e. a change of 0.447 in men’s reports of sexual IPV in South Africa, 0.375 reduction in women’s reports of severe IPV in Ghana, and a reduction in physical and sexual IPV of 0.368 and 0.339 in Nepal.

In order to examine evidence that the reduction in violence reported stemmed from repeated assessments in the research process, we compared the change in the cohort studies with the repeat cross-sectional studies. For cohorts, 7/18 assessments (39%) showed an increase in reporting of violence between baseline and midline and in 11/18 (61%) there was a decrease. In 4 of 9 women’s cohort assessments, there was an increase at midline, and in 3 of 9 male cohort assessments. In three of 12 cohort studies, with three data points, the endline was greater than the midline, and in 6/14 cohort assessments with three data points, the endline was greater than the baseline. In 5/8 women’s cohort assessments, the endline was higher than baseline, and this did not occur in any of the male cohorts. Only one of the repeat cross-sectional studies had a midline, this was Nepal where all assessments were lower at midline than baseline, and higher at endline than at midline. Across the repeat cross-sectional studies, in 1 of 9 female assessments, endline was higher than baseline, in 8/9 it was lower, but we cannot attribute this to the Hawthorne effect as the participants had not been interviewed previously.

### Mean absolute and proportionate change

Table 3 presents the mean change seen in absolute percentage points and proportions across the studies. Across all the studies, notwithstanding different IPV measures and two genders, the mean difference between baseline and endline was 7.01 percentage points, a mean proportionate change of 17.5%. However, the change between baseline and midline, or midline and endline was sometimes larger than between baseline and endline and the largest difference was on average 8.39 percentage points (a 0.216 proportionate difference).

**Table 3.** Mean change in the control arms of across studies, all types of IPV and genders

	Mean change in percentage points baseline – endline (95%CI)	Mean largest change in percentage points between two time points (95%CI)	Mean proportionate change (baseline to endline)	Mean largest change as a proportion of baseline
All studies (12 datasets)	7.01 (3.68, 10.33)	8.39 (5.08, 11.70)	0.175	0.216
All studies with no active control arms (10 datasets)	3.21 (1.59,4.83)	4.88 (0.94, 8.83)	0.134	0.177
Cohort studies with no active control arms (5 datasets)	4.00 (0.38, 7.62)	6.29 (0, 17.04)	0.13	0.171
Repeat cross-sectional (5 datasets)	2.47 (0, 5.56)	3.57 (0,10.22)	0.137	0.182

Excluding the Zambia study with its active control made a substantial difference to the mean difference between baseline and endline, which was then 3.21 (range 0.5–6.8), compared to overall (7.01 (range 0.5 to 36.5)). The table shows that the greatest mean difference in baseline to endline was in the studies with cohorts, rather than repeat cross-sectional samples (4.00 versus 2.47 percentage points), with the largest assessed change being, on average, 6.29 (cohorts) and 3.57 (repeat cross-sectional) percentage points. There was very little difference between mean proportionate change between baseline and endline across study types, 0.13 (cohort) and 0.137 (repeat cross-sectional). The average of the largest proportionate changes was 0.171 (cohorts) and 0.182 (repeat cross-sectional).

More women than men reported physical and severe IPV at baseline, across all studies. The difference was fairly small in Zambia for physical IPV and in Ghana for severe IPV, but it was not small in other assessments (Table 2). In Ghana, men reported more sexual IPV than women did, but women reported more sexual IPV in both samples in Rwanda and Zambia, and in South Africa, there is no difference in sexual IPV reports between men and women.

### Mean absolutely and proportionate change by IPV type and gender

Across all studies, the average number of percentage points difference between baseline and endline, whether reflecting a reduction or increase in IPV reports, and proportionate change, was greater for men than women for physical IPV (Table 4), but if the Zambia study is excluded from the analysis, the differences by gender were very small. The pattern was different for sexual IPV, with more variation in terms of absolute percentage points difference between baseline and endline in women’s reports than in men’s. However, when these are analysed as a proportion of the baseline level, there was proportionately more variation in men’s reports, with a notably wide range seen, especially when the largest change between two points is considered. For severe IPV, the pattern shows men on average reporting a greater difference between baseline and endline than women, but the average of the largest difference between points is a little greater for women than men.

**Table 4.** Change in the control arms of different study types, by type of IPV and gender

	Gender	Change in % points baseline – endline (mean)	Range	Largest change in % points between two time points (mean)	Range	Proportionate change baseline to endline (mean)	Range	Largest change as a proportion of baseline (mean)	Range
**Physical IPV**									
All studies*	Female	6.70	0.9,27.8	8.06	1.0,27.8	0.162	0.024,0.37	0.201	0.024,0.37
	Male	8.36	1.7,25.0	9.02	1.7,25.0	0.203	0.076, 0.336	0.216	0.076, 0.336
All studies with no active controls ^@^	Female	3.18	0.9, 5.7	4.77	1,10.8	0.127	0.024, 0.289	0.173	0.024, 0.368
	Male	4.20	1.7, 6.8	5.03	1.7,9.7	0.17	0.077, 0.298	0.186	0.077,0.298
Cohorts with no active controls ***	Female	3.10	0.9, 5.7	5.23	2.2, 10.8	0.078	0.027, 0.113	0.12	0.066, 0.180
	Male	6.60	6.4, 6.8	8.25	6.8,9.7	0.213	0.127, 0.298	0.245	0.192, 0.298
Repeat cross-sectional***	Female	3.27	1, 4.4	4.30	1.0, 7.5	0.176	0.024, 0.288	0.227	0.024, 0.368
	Male	1.80	1.7, 1.9	1.80	1.7, 1.9	0.127	0.076, 0.178	0.127	0.076, 0.178
**Sexual IPV**									
All studies^$^	Female	5.60	0.5, 23.1	6.55	0.9, 23.1	0.134	0.013, 0.332	0.175	0.024, 0.339
	Male	3.32	0.6,5.0	6.04	0.6, 16.8	0.14	0.036, 0.238	0.229	0.034, 0.553
All studies with no active controls **	Female	2.10	0.5, 5.2	3.24	0.9, 8.1	0.094	0.013, 0.172	0.144	0.024, 0.339
	Male	2.9	0.6, 4.0	6.3	0.6, 16.8	0.146	0.036, 0.238	0.258	0.034, 0.553
Cohorts with no active controls ^#^	Female	2.85	0.5, 5.2	2.1	0.9, 3.3	0.093	0.013, 0.172	0.067	0.024, 0.109
	Male	3.6	3.2, 4.0	10.4	4.0, 16.8	0.155	0.105, 0.204	0.378	0.204, 0.553
Repeat cross-sectional ***	Female	1.6	0.9, 2.2	4	1.7, 8.1	0.095	0.038, 0.200	0.137	0.048, 0.339
	Male	2.2	0.6, 3.8	2.2	0.6, 3.8	0.137	0.036, 0.238	0.196	0.036, 0.238
**Severe IPV**									
All studies*	Female	7.99	0.9, 36.5	9.91	1.2, 36.5	0.189	0.022, 0.552	0.240	0.024, 0.552
	Male	10.08	1.7, 34.7	10.66	1.7, 34.7	0.228	0.086, 0.529	0.240	0.086, 0.529
All studies with no active controls **	Female	3.23	0.9, 6.6	5.48	1.2, 11.1	0.129	0.022, 0.167	0.189	0.024, 0.375
	Male	3.93	1.7, 5.9	4.65	1.7, 8.8	0.152	0.086, 0.254	0.168	0.086, 0.254
Cohorts with no active controls ***	Female	3	0.9, 5.3	5.43	2.4, 11.1	0.094	0.022, 0.167	0.141	0.058, 0.198
	Male	5.8	5.7, 5.9	7.25	5.7, 8.8	0.191	0.127, 0.254	0.222	0.189, 0.254
Repeat cross-sectional***	Female	3.47	1.2, 6.6	5.53	1.2, 8.8	0.163	0.024, 0.375	0.236	0.024, 0.375
	Male	2.05	1.7, 2.4	2.05	1.7, 2.4	0.114	0.086, 0.141	0.114	0.086, 0.141

* 12 datasets **9 datasets ***5 datasets ^$^ 11 datasets ^#^ 4 datasets ^@^ 10 datasets.

When we just consider the cohort studies (excluding Zambia), for physical IPV, there was quite a large difference in the percentage points change and proportionate change with much more variation in the reports of men than women. With the repeat cross-sectional studies, the pattern was the opposite, with more variation in physical IPV reports, absolute percentage points and proportionate change found among women than men. Among our studies, there was a larger variation in actual percentage points for cohort than cross-sectional studies, and this was strongly influenced by changes in men’s reports of physical IPV; however, there is very little difference in the average proportionate change by study design. For sexual IPV there was very little difference between gender and by study design type, except in one cohort study (in South Africa) where men had a large difference from baseline to midline. Much of the same pattern was seen for severe IPV (excluding Zambia) where only the men’s cohort studies were somewhat different in their absolute percentage point change and largest change, from women and the repeat cross-sectional studies. The proportionate changes, however, did not differ very much by study type.

## Discussion

We have shown that among the studies in *What Works* repeated assessments of the control population, whether a closed cohort or identified through repeat cross-sections of the community, showed an average absolute variation of 3.21 (95%CIs 1.59, 4.83) percentage points for the studies with no active controls. However, the magnitude of this variation seems much larger when viewed as a proportionate change; average proportionate change in the control arm between baseline and endline was 0.177, which is close to a 20% difference. More concerning was that 10 assessments, from 4 of the 7 studies, showed a proportionate change between baseline and endline, or midline and baseline/endline in excess of 30% among control participants. The changes were largest in the study with an on-going active control arm, which focused on safety monitoring with weekly phone calls to control arm participants by a research assistant with a safety protocol. But very large proportionate changes were also found in some other studies, with no active control. The large changes seen in control arms show that we very much need control arms in the field of VAW prevention to avoid erroneous evaluation findings.

We considered whether the changes in the control arms might be due to genuine changes resulting from, for example, new laws, poverty reduction programmes, or even spill over from the intervention. We have discounted this. The time between measurements was relatively short for structural changes to have an impact, the direction of change was inconsistent, we are not aware of major new laws and programmes coming on stream in the study areas, and we have enough knowledge of what is required to change men’s use of violence to consider the risk of a small amount of spill over (had it occurred) resulting in a measurable effect in quite a large study population to be very small indeed [1,26].

The control arm fluctuations observed across these studies increase the possibility of both type 1 and 2 errors (spuriously identifying impact of an intervention, or failing to identify a truly beneficial intervention as effective, respectively) and have important implications for study power. The risk is greatest where there are fewer measurements over time, limiting the ability to consider trends, and are especially acute when only two measures (baseline and endline) are present. An important example of a trial in the VAW field that may have been affected by such issues is the SASA! evaluation, which concluded that ‘the intervention was associated with … lower levels of past year experience of sexual IPV (0.76, 95% CI 0.33 to 1.72)’ but the intervention baseline and endline prevalence were 13% and 14%, and the control arm prevalence measures were 11% and 20%. Mathematically, this conclusion was clearly driven by the high prevalence of sexual IPV in the control arm at endline, which dramatically raises the risk of a Type 1 error or false positive [27]. It is very important that the VAW intervention evaluation field becomes more conscious of this possibility and accounts for it in study design, data analysis and interpretation. Considerably more defence against possible Type 1 errors can be provided by having more measures of VAW (i.e. not just focused on physical and sexual IPV, but including emotional and economic IPV, or non-partner sexual violence) and looking at trends across measures with the outcome determined by a pre-specified pattern of change across multiple measures and time points.

Our findings have important implications for sample size calculations as these are normally specified based on an anticipated baseline prevalence, but with an implicit assumption that change will only be found in the intervention arm (if there is an impact from the intervention). We have shown that some fluctuation in the prevalence in the control arm in VAW research is routinely encountered and we conclude that it should be therefore be taken into account in the sample size calculation. This will require proportionately larger studies and more resources per study but the impact on evaluation research funds in the field could be mitigated by raising the bar for commencement of RCTs until after all the design and implementation issues for interventions have been ironed out through iterative mixed methods research in pilot evaluations, and estimated effect sizes for populations of interest are available.

In this analysis, we considered whether the change in the control arms could be due to the Hawthorne effect or another repeat interview effect (perhaps changes as a result of completing the questionnaire). We have concluded that there is little evidence that this is the case as the studies that more consistently showed incrementally reducing the prevalence of violence in the control arm where the repeat cross-sectional studies were individual participants were not given multiple interviews. The pattern with the cohort studies was very mixed (with changes going upwards and downwards), suggesting random directional changes, although we are viewing all the data at the pooled level and not at an individual participant level, where possibly there could have been other influences.

We assume that control arm change we have discussed is non-differential misclassification as most sources of differential misclassification that we are concerned with in RCTs are due to the intervention. Analysis by gender suggests that non-differential misclassification was probably a bigger problem for men than women, as the change between time points in men’s reports was in general larger than that for women. An important perspective of men’s reports versus women’s comes from the couples studies. In Rwanda, the men and women of couples showed similar patterns of reporting – and most notably a high consistency in their reports. However, the actual levels reported by men were a third to a half those reported by women. In Zambia, men and women’s reports of physical IPV were very consistent, but sexual IPV at baseline was reported by many fewer men than women, and severe IPV was reported by notably fewer men. At endline in Zambia, there was still a difference in reports of sexual and severe IPV, but it was much less marked. A greater unreliability in men’s perpetration versus women’s experience reports of VAW was seen in the study on test–retest reliability in South Africa, which found that measures (the same as those used in these studies) were fairly reliable at a group level but less so at an individual level with male reports less reliable than women’s, especially of past year sexual violence [12]. None of the measures in terms of test–retest reliability were more than ‘moderately’ reliable (Kappa <0.6) and the repeat interviews were completed 2 weeks apart. It is not clear whether the key problem here is social desirability bias or just that men are often not encouraged to reflect on their own behaviour. They may lack the self-awareness and/or situational awareness to correctly answer some of the commonly used questions about especially sexual IPV. For example, men may not understand (or even notice) when a woman they are having sex with is frightened/threatened and feeling coerced, which is clearly a huge problem in and of itself.

In repeat cross-sectional studies, given the fact that we have different people interviewed at every round we would expect misclassification to be a bigger problem than cohort studies, but we did not actually see this pattern. There seemed to be if anything a larger problem in repeatability of the control assessment for the cohort studies. We investigated to see if this was due to the impact of the study from South Africa, which had a smaller sample size than the other studies, apart from Zambia, but did not show that excluding South Africa reversed the pattern.

We recognise that from the perspective of women experiencing VAW, all success in reducing their exposure is valuable. However, at a population level, we need to understand the magnitude of reduction in VAW that an intervention should achieve to be considered ‘clinically significant’. In this respect, we would refer to the magnitude of reduction where we have some confidence this could only be achieved through an effective intervention and would be almost certainly outside the realm of non-differential misclassification. Whilst it is not possible to conclusively identify this point from our data, our findings certainly suggest that it would be necessary for an intervention to see an absolute percentage point change of ± 7% in the absence of any active control, and one would require considerable caution in interpretation of positive findings if the proportionate change was less than 0.4, especially in studies with a baseline prevalence below 20%. In the light of our findings around control arm changes, we recommend three or preferably more data points for studies.

Notwithstanding these findings, we recognise that RCTs are not appropriate for new interventions or interventions where there has not already been extensive monitoring and evaluation with course correction, as indicated. There is a considerable risk, felt more acutely in our relatively young field of research with scarce resources like the VAW prevention intervention evaluation field, that energy will be diverted too soon into determining if an intervention works via an RCT, rather than using more informative and rapid means of gathering information on how it may or may not be impacting on people’s lives. Strong mixed methods designs are a good alternative for newly developed interventions and provide information in a quicker and more accessible manner as shown in research in What Works, notably our work in Tajikistan [28]. A consideration in such research is whether it is strengthened by having a small control arm; however, our findings from the RCTs would caution against this as the problem of non-differential error is greater in small studies. We would encourage researchers to recognise that control arm changes and comparisons can mislead.

A further implication of the large fluctuations in control arm prevalence due to non-differential misclassification is that we would need as a field to be very cautious about concluding that VAW prevention interventions may show signs of harm. There has been an understandable hesitation to discuss this thus far in the literature, even when occasional statistically significant results in the unanticipated direction are found [1]. Our findings support the wisdom of such a position. We suggest that the circumstances in which an intervention could be regarded as harmful should include there being a plausible mechanism for the harm proposed and that standards for judging harm should be as rigorous for those used to assess benefit.

This study had a huge strength of being able to reflect on control arm changes across multiple studies from different geographical settings where the research was based on the same measures of VAW. This has enabled reflections to extend beyond bias due to measurement differences, to shifting reporting due to non-differential misclassification in control arms. There are a number of limitations. The number of studies was still small and we have focused on reported percentage change without much statistical analysis as the data in many respects very limited. In particular, we have largely not presented 95% CI as they are not very meaningful with a small number of observations. We have focused on across trial summary changes and do not take into account differences in contributions to the results based on sample size differences. Nor do we have data on individual level change. The analysis is fairly rudimentary, but we believe it is sufficiently robust to enable us to make the key points of this paper, which are very important for the VAW prevention research methods field.

## Conclusion

Analysis of control arm change in the What Works studies supports the importance of having a control arm in research conducted to detect whether an intervention has an ability to meaningfully reduce VAW, but we have found considerable potential for type 1 and type 2 error if trials are not appropriately designed and conducted. We perceive that most of the changes observed in the control arm are due to non-differential misclassification, rather than systematic bias as we did not see effects clearly attributable to multiple assessments, the Hawthorne effect, gender, or even due to repeat cross-sectional studies rather than cohort studies. Nonetheless, changes in control arms are very important for understanding VAW intervention impact as measures of VAW in control arms seem to substantially fluctuate over time.

We recommend that anticipated change in control arms be routinely taken into account in sample size calculations to ensure that there is adequate power, that multiple (3+) assessments be a standard in RCT design and appropriately funded, and that a priori rules for determining the effect of interventions be framed around the change in multiple measures of VAW with some evidence of change in other outcomes, a proportionate change of 0.4 (especially where the baseline prevalence is <20%) and absolute change of more than 7 percentage points. Further whilst recognising the importance of RCTs in ultimately showing effect, we caution against their use with interventions that have not been previously evaluated with mixed methods, improved, and shown to be sufficiently rigorously designed and implemented to be able to substantially advance our knowledge and understanding in the field when evaluated using an RCT.

## Paper context

Control arm change in RCTs is rarely examined. Our secondary analysis of data from the control arms of evaluations of violence against women and girls’ prevention programming with comparable measures shows a small difference in absolute percentage points difference between assessments, but often large (over 30%) proportionate change due to measurement error. This is important for sample size calculations and in determining trial effect. We recommend more elaborate pre-defined outcomes for determining intervention impact.
